# Squared Focal Intensity Distributions for Applications in Laser Material Processing

**DOI:** 10.3390/ma14174981

**Published:** 2021-08-31

**Authors:** Henrike Schlutow, Ulrike Fuchs, Frank A. Müller, Stephan Gräf

**Affiliations:** 1 Asphericon GmbH, Stockholmer Str. 9, 07747 Jena, Germany; h.wilms@asphericon.com (H.S.); u.fuchs@asphericon.com (U.F.); 2 Otto Schott Institute of Materials Research (OSIM), Friedrich Schiller University, Löbdergraben 32, 07743 Jena, Germany; frank.mueller@uni-jena.de

**Keywords:** beam shaping, squared top-hat, fs-laser, laser surface structuring, laser-induced periodic surface structures, micro-channels

## Abstract

Tailored intensity profiles within the focal spot of the laser beam offer great potential for a well-defined control of the interaction process between laser radiation and material, and thus for improving the processing results. The present paper discusses a novel refractive beam-shaping element that provides different squared intensity distributions converted from the Gaussian output beam of the utilized femtosecond (fs) laser. Using the examples of surface structuring of stainless-steel on the micro- and nano-scale, the suitability of the beam-shaping element for fs-laser material processing with a conventional f-Theta lens is demonstrated. In this context, it was shown that the experimental structuring results are in good agreement with beam profile measurements and numerical simulations of the beam-shaping unit. In addition, the experimental results reveal the improvement of laser processing in terms of a significantly reduced processing time during surface nano-structuring and the possibility to control the ablation geometry during the fabrication of micro-channels.

## 1. Introduction

In modern laser material processing, beam shaping is receiving growing attention, as it allows the focal intensity distribution to be adjusted to the interaction process between the laser radiation and the material [1,2,3]. This makes laser processing even more flexible, precise and effective. Moreover, the range of achievable types of structures is extended [4,5,6] and surface properties can be adjusted in a targeted manner [7,8,9]. Top-hat profiles are characterized by a uniform intensity distribution over the beam cross-section in a specific working plane. They can be generated by a large variety of available beam-shaping techniques, including apertures [10], refractive [11,12,13] and diffractive optical elements [10,14], as well as beam integrators or homogenizers [10,15]. Finally, a spatial light modulator provides a flexible optical device that allows to create various intensity profiles based on the dynamical modification of the amplitude, phase and polarization of the incoming laser beam [16,17,18].

The advantage of refractive beam shapers is provided by their simple structure and a very high conversion efficiency. They are less sensitive to wavelength changes, easier to manufacture and can withstand higher laser powers when compared to diffractive elements. Therefore, the present work aims at the introduction of a highly flexible and compact refractive, freeform beam-shaping element that is suitable for laser material processing applications.

Large-area structuring of surfaces on the micro- and nano-meter scale usually requires scanning of the focused laser beam across the material surface. In the case of the commonly used Gaussian intensity distribution, a certain inhomogeneity of the deposited energy occurs, which in turn has an influence on the processing result. A first improvement was demonstrated in this context by using a beam shaper that allows to generate a rotationally symmetric top-hat profile [19]. However, due to the rectangular symmetry of the (unidirectional) scan procedure with a specific overlap of the adjacent scan lines, the use of a squared top-hat seems to be even more advantageous with respect to homogeneous energy coupling. This squared top-hat is derived from the collimated Gaussian intensity profile of the input beam by combining the investigated refractive beam shaper with a focusing optics. The latter can be a simple focusing lens as well as a complex f-Theta objective, as is very often used for laser-based surface structuring. Therefore, a main objective of this study was to evaluate whether the x-y-symmetry of the focal distribution can further improve material processing when working with a scanner and an f-Theta lens. This aspect plays an essential role with regard to modern laser systems and their high repetition rates, whose maximum utilization ensures high processing efficiency. For this purpose, the beam-shaping element was practically tested in different applications on stainless-steel as a substrate material. This includes surface nano-structuring in the form of laser-induced periodic surface structures (LIPSS), which allow to engineer surfaces with versatile functional properties [7,20]. Furthermore, the ablation-based micro-structuring of the sample surface using femtosecond lasers (fs-lasers) was demonstrated as a conventional processing technique [21].

## 2. Materials and Methods

### 2.1. Focal Beam-Shaping and Characterization

The most common approach for shaping focal intensity distributions is based on Fourier optics, where the focusing optics transforms the incoming beam profile into the desired shape. This is well-explained for rotational symmetry in [19,22]. However, for non-rotationally symmetric focal intensity distributions, the symmetry of the beam-shaping element needs to be broken, while the focusing optics remains the same. Therefore, the beam-shaping element becomes a freeform surface. As a phase plate, it imprints a phase modulation on the incoming plan wave of the collimated beam, which stays collimated as this modulation is in the order of λ (PV) and thus very small. The focusing optics performs the Fourier transformation of this modified wave front and therefore determines the width of the generated profiles inverse proportional to the numerical aperture (NA). Consequently, this approach is modular with regards to the focusing optics. The phase plate (a|SqAiryShape) itself is a freeform distribution based on a super Gaussian profile:

(1) z(h)=He−2(4h2D2)M2

of order M = 8, diameter D and plateau height H in x-y-symmetry. The element is made of fused silica via CNC-based grinding and polishing. It can therefore withstand high-power laser intensities as well as ultrashort laser pulses, as employed in the experimental studies of the present work.

As a measure of how much the beam shaping will be affected by diffraction effects, the β-factor:

(2) $$\beta =\frac{2\pi \mathrm{RD}}{f_{L}\lambda }$$

can be used [23], with R being the half diameter of the entrance beam, D being the half diameter of the focal distribution, f_L_ being the focal length and λ being the wavelength. Using the specific parameters of the present study (R = 10 mm, D = 25 µm, f_L_ = 100 mm and λ = 1030 nm) leads to β = 15.25. Having such a large value ensures stable beam-shaping profiles, which are essential for well-controlled material processing.

**Figure 1:**
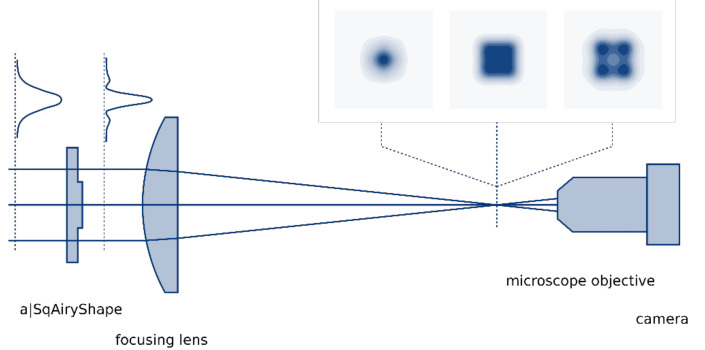
Experimental setup for characterization of the freeform beam-shaping element.

The squared focal intensity distribution was characterized in order to compare the experimental results with simulations. For this purpose, a supercontinuum laser (SC-400-4, Fianium, Southampton, UK) was used as a light source, from which λ = 1064 nm was selected via a spectrometer (LLTF Contrast, Fianium, Southampton, UK), coupled to a fiber, collimated (ACM25-20-L-C-1064, asphericon, Jena, Germany) and expanded (BAM25-175-D-C-1064, asphericon, Jena, Germany) to a collimated beam with 10 mm diameter. This beam fulfilled the input conditions for the freeform beam-shaping element. All following optical components used for characterization of the beam shaping are depicted in Figure 1. The beam-shaping element (a|SqAiryShape) was placed in the collimated beam, which was subsequently focused by a plano-convex lens with 300 mm focal length (#38-631, Edmund Optics, Mainz, Germany). The focal region was then imaged onto a camera (SP928, Ophir, Germany) by using a microscope objective (Aspheric Lens Kit 5720-B, Newport, Irvine, CA, USA). The setup was calibrated to obtain the real physical size of the focal distribution.

In the simulation, only the freeform beam-shaping element and the plano-convex focusing lens were considered. The focal intensity distribution was calculated via the point spread function with CodeV (Synopsis). This calculation is based on the squared Fourier transform of the pupil function [24]. The latter includes all aberrations and thus the phase manipulation introduced by the beam-shaping element.

**Figure 2:**
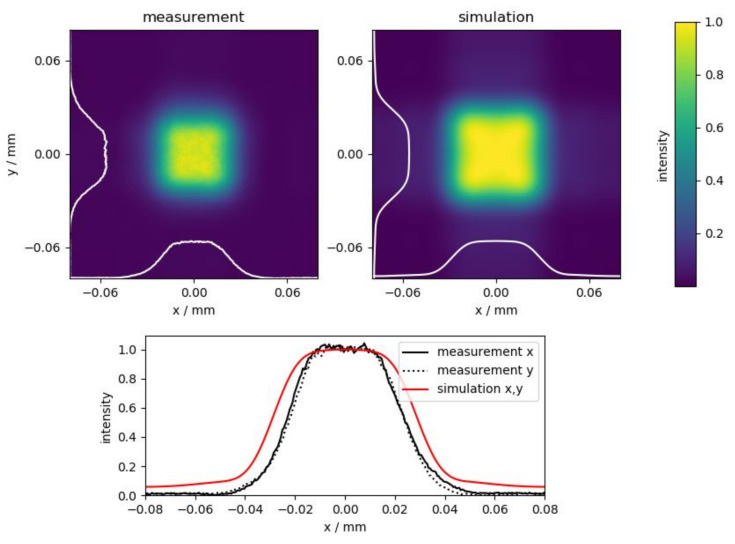
Color maps and cross-sections of the squared focal intensity distribution of the top-hat closest to the focal plane (λ = 1064 nm, f = 300 mm) normalized to the maximum intensity for comparison between measurement and simulation.

Close to the focal point, various intensity distributions, such as a squared top-hat or squared donut, emerge. The following laser processing experiments were carried out with the squared top-hat, which occurs closest to the focal point. This is the top-hat shown in Figure 2. Comparison with the simulation, especially through the cross-sections in the bottom of Figure 2, reveals close agreement between measurement and simulation in terms of shape and size of the focus spot.

**Figure 3:**
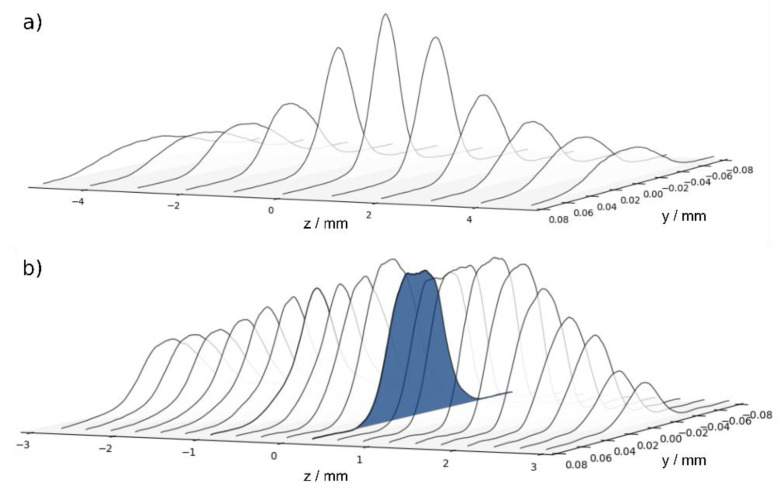
Profile cross-sections measured along the optical axis (z) in the focal region (**a**) without and (**b**) with freeform beam-shaping element.

A sequence of those cross-sections taken along the optical axis is plotted in Figure 3. The upper panel (Figure 3a) shows the beam waist of the Gaussian input beam without the freeform beam-shaping element. With the beam-shaping element (panel b), the Gaussian distribution is notably flattened to a top-hat. Note that the beam shaping only occurs close to the focal point (z = 0 mm), whereas the profile remains almost Gaussian for larger distances. The blue-shaded distribution corresponds to the one shown in Figure 2. 

### 2.2. Material Processing Setup and Sample Preparation

**Figure 4:**
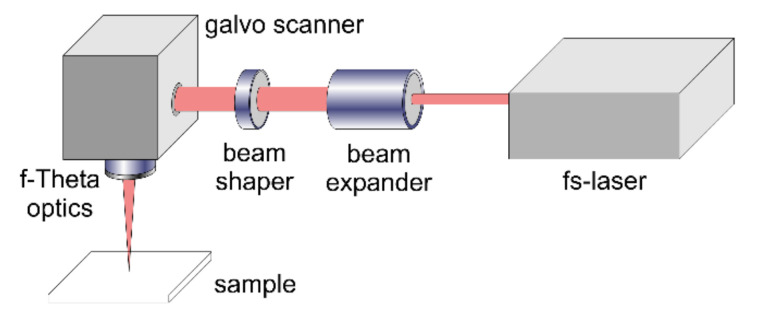
Experimental setup used for surface structuring experiments.

The operation of the beam-shaping element was investigated by means of surface structuring experiments using the experimental setup illustrated in Figure 4. The linearly polarized Gaussian output beam (M^2^ ~ 1.08) of the diode-pumped Yb:KYW thin-disc fs-laser system (JenLas D2.fs, Jenoptik, Germany) with a central wavelength λ = 1025 nm is characterized by a pulse duration τ = 300 fs (FWHM) and pulse energies E_imp_ ≤ 40 µJ at a repetition frequency f_rep_ = 100 kHz. Before passing the beam shaper, the output beam was widened by a beam expander (5×). The shaped beam was then focused onto the sample surface by a galvanometer scanner (IntelliScan14, Scanlab, Puchheim, Germany). The focal length of the associated f-Theta objective (JENar, Jenoptik, Jena, Germany) was f_L_ = 100 mm.

The structuring experiments were carried out on commercially available austenitic stainless-steel (X2CrNiMo17-12-2, Outokumpu, Krefeld, Germany) at normal incidence and under air atmosphere. Prior to laser irradiation, the sample surface was manually grounded and polished to a mirror finish, with an average surface roughness R_a_ ~4 nm. This procedure involves the use of SiC abrasive paper of 800, 1200 and 2400 grit and 6, 3 and 1 µm polycrystalline diamond suspension, respectively. Besides, the sample surfaces were ultrasonically cleaned in acetone and isopropanol both before and after laser structuring. The morphology of the samples was characterized by scanning electron microscopy (SEM) (SigmaVP, Carl Zeiss, Oberkochen, Germany) at an accelerating voltage of 5 kV using a secondary electron detector. The spatial periods and the orientation of the LIPSS were quantified by 2D-Fourier transform analysis (2D-FT) of the SEM micrographs. White light interference microscopy (WLIM) (CCI HD, Taylor Hobson, Weiterstadt, Germany) was utilized to evaluate the surface topography. The microscope was equipped with a 50× objective.

## 3. Results and Discussion

### 3.1. Single-Spot Experiments

The first part of the experiments was to investigate whether the theoretical calculations for the focal intensity distribution correlate with the geometry of the ablated region within the focal spot on the material surface. For this purpose, the surface was irradiated with 10 single fs-laser pulses with a temporal pulse spacing of 10 µs (f_rep_ = 100 kHz) and a single-pulse energy of E_imp_ = 6.5 µJ. Without the beam-shaping element in the beam path, the utilized f-Theta objective results in a focal spot diameter of 2w_f_ = (24 ± 0.5) µm, determined using the method of Liu [25]. This corresponds to a fs-laser peak fluence of F = 2E_imp_/(πw_f_^2^) = 2.9 J/cm^2^ at the pulse energy used.

**Figure 5:**
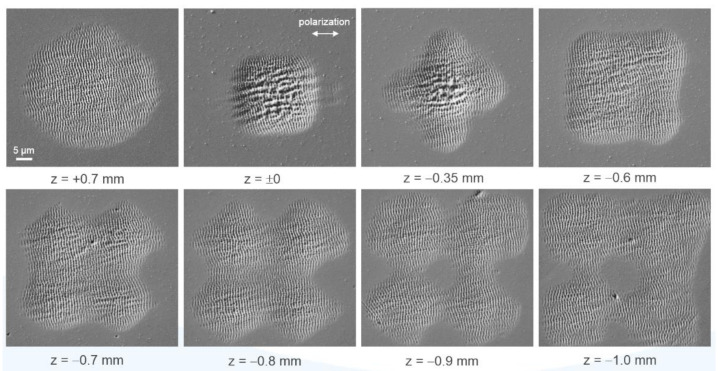
SEM micrographs of the stainless-steel surface after irradiation of 10 linearly polarized single fs-laser pulses at different z-positions of the sample surface within the focal intensity distribution. For comparability, the single-pulse energy was kept constant at E_imp_ = 6.5 µJ.

**Figure 6:**
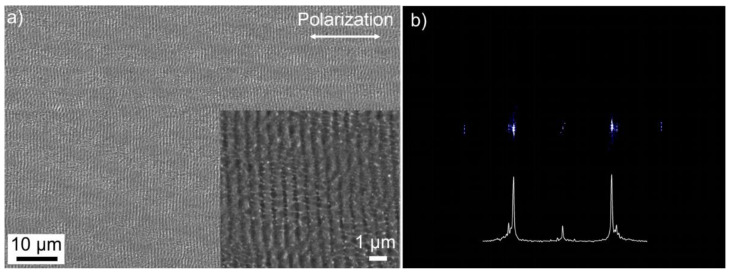
LIPSS structuring of stainless-steel surfaces using the squared top-hat profile (z = 0 mm) of the linearly polarized fs-laser beam (2w_f_ ~30 µm, E_imp_ = 3.5 µJ, v = 1 m/s, Δx = 10 µm): (**a**) SEM micrograph and (**b**) 2D-FT spectrum and its corresponding cross-section calculated from the SEM micrograph quantifying the homogeneity and orientation of the LIPSS.

Figure 5 shows SEM micrographs of the ablation spots obtained at different distances (z-positions) of the stainless-steel surface from the focusing objective using the freeform beam-shaping element. At the reference position (z = 0 mm) and at z = −0.6 mm, squared top-hat intensity profiles are available that result in square material ablation of size (30 × 30) µm^2^ and (40 × 40) µm^2^, respectively. As described later, these intensity profiles are predestined for numerous laser material processes that are based on scanning procedures with x-y-symmetry (Figure 6). Assuming a perfectly rectangular fluence distribution of both top-hat profiles, the larger focal dimensions together with the pulse energy E_imp_ = 6.5 µJ result in a lower fs-laser peak fluence of F = 0.72 and F = 0.41 J/cm^2^ respectively, when compared to the initial Gaussian focal spot. It should be noted that the peak fluence, F, is identical to the average fluence, F_av_, of the fluence distribution. The larger fluence at z = 0 compared to z = −0.6 mm is indicated by the stronger melt formation in the ablation spot fabricated with the smaller top-hat profile.

In addition to the top-hat profiles, the beam-shaping element provides other intermediate stages, which are characterized by more exotic intensity distributions. At z = −1.0 mm, a donut-shaped focal intensity distribution with square beam cross-section can be observed, characterized by the zero-intensity center on the beam axis. In contrast, at z = −0.35 mm, the intensity distribution is almost completely reversed, which is why a stronger intensity can be observed at the center. Both examples are intensity distributions suitable for the generation of novel surface structures (e.g., bio-inspired surfaces) [5].

The SEM micrographs of the single-spot experiments reveal a well-defined modulated surface topography within the ablation crater, whose periodicity is in the sub-µm range and therefore much smaller when compared to the diffraction-limited focal spot diameter of a few tens of µm. These periodic patterns are termed laser-induced periodic surface structures (LIPSS), which for metals are typically oriented perpendicular to the linear beam polarization [26]. The most widely accepted theory describes the formation of LIPSS as a result of interference of the incident laser radiation with surface electromagnetic waves generated by scattering at the nano-rough surface [27,28]. These scattering processes are possible despite the polished initial surface, since the LIPSS formation process is generally a multi-pulse process. 

As Figure 5 shows, the exact morphology of the LIPSS pattern within the focal spot depends on the fluence distribution in the beam cross-section, i.e., on the intensity profile. A particular contribution is provided by melt formation in the intensive regions of the beam cross-section. However, it becomes evident that a particularly homogeneous LIPSS distribution can be realized by the appropriate choice of the z-position, which suggests here a constant fluence in the beam profile. This is a key aspect for homogeneous large-area structuring with LIPSS [29]. In addition, the possibility to control the LIPSS distribution within the focal spot by choosing a certain beam profile leads to an extension of the current state-of-the-art and, thus, to a higher flexibility of the direct-writing structuring method. The fluence values calculated above for the top-hat profile are comparable with the fluence required for ablation-based LIPSS formation on stainless-steel at comparable irradiation parameters [30,31].

### 3.2. Laser Surface Structuring on the Nanoscale

Based on the demonstrated LIPSS formation within the focal spot, the nanostructures can be scaled to large surface areas by a relative movement between the focused fs-laser beam and the sample surface. This is particularly achievable with metals due to the linear absorption of the fs-laser radiation. In this context, the specific optical properties make stainless-steel a well-suited candidate for the production of highly regular LIPSS [31]. This concerns in particular the exact alignment and a small dispersion of the spatial frequencies of the LIPSS, which are coherently linked to each other despite the unidirectional scanning procedure [30,31].

The SEM micrograph in Figure 6a shows the stainless-steel surface obtained from scanning the squared top-hat profile (z = 0 mm) with a scan speed of v = 1 m/s and a line spacing of Δx = 10 µm across the sample surface. The single-pulse energy was reduced to E_imp_ = 3.5 µJ, i.e., to F = 0.39 J/cm^2^, in order to avoid the extensive melt formation observed in Figure 5. The calculated 2D-FT spectrum (Figure 6b) confirms the high regularity of the LIPSS aligned perpendicular to the linear beam polarization by the very narrow characteristic peaks. The spatial periods of the LIPSS were determined to be around (950 ± 20) nm. 

**Figure 7:**
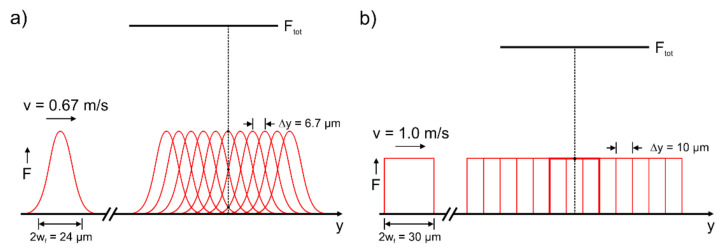
Overlap and contribution of individual pulses during scanning of a single line using (**a**) the Gaussian focal intensity distribution and (**b**) an ideal top-hat beam.

The obtained regularity is comparable with our previous work on rotationally symmetric beam shaping [19], although the following main differences should be discussed:

(1) As already demonstrated in [30,31], highly regular LIPSS can also be generated with conventional Gaussian beam profiles. However, the radial intensity dependence and the circular spot area require a larger overlap of the laser pulses in both x- and y-directions to realize an almost homogeneous energy deposition. The optimal process parameters for Gaussian beam processing were derived in [19] to be v = 0.67 m/s, Δx = 6 µm, E_imp_ = 2.6 µJ, F = 1.15 J/cm^2^ at f_rep_ = 100 kHz and 2w_f_ = 24 µm. These values result in a processing rate of about 32 s/cm^2^ and in a laser pulse number N_eff_2D_ = (πw_f_^2^·f_rep_)/(v⋅Δx) ~11.3, that effectively hit the focal spot area [28].
(2) The main difference between the ideal squared top-hat and the Gaussian profile results from the relative movement during scanning. Figure 7a illustrates that for the Gaussian profile at a certain position y at the surface, every single pulse of the total required number (N_eff_2D_) contributes to structuring with different fluence values. Consequently, the specific fluence of some of the individual pulses can be significantly smaller than the ablation and LIPSS formation threshold of the material.
(3) In the case of the top-hat profile (z = 0) with its square shape, the slightly larger beam diameter (here given by the beam width in x- and y-directions) and steeper flanks, LIPSS structures with almost equal properties can be achieved. Nevertheless, the structuring can be performed with larger scanning speed (v = 1 m/s) and line separation (Δx = 10 µm). As described above, the pulse energy must be increased slightly for this to exceed the required LIPSS formation threshold. The optimum parameters, however, result in N_eff_2D_ = 9 and a processing rate of about 14 s/cm^2^, which is more than a factor of 2 faster than structuring with the conventional Gaussian beam.
(4) The accumulated fluence of the ideal top-hat profile with F_tot_ = N_eff_2D_·F = 3.51 J/cm^2^ is smaller than for the Gaussian intensity distribution (F_tot_ = 6.47 J/cm^2^), since during structuring with the top-hat, each single pulse contributes equally to F_tot_ (Figure 7b).

### 3.3. Surface Micro-Structuring

In order to investigate the influence of the intensity distribution on the micro-structuring of surfaces, channels were created using the top-hat profile (z = 0 mm) and 10 over-scans with v = 0.1 m/s. Earlier studies with rotationally symmetrical top-hat and donut-shaped beam profiles already showed that steeper channel walls and a better surface quality (especially at the bottom) can be achieved in comparison to the Gaussian profile [19]. 

**Figure 8:**
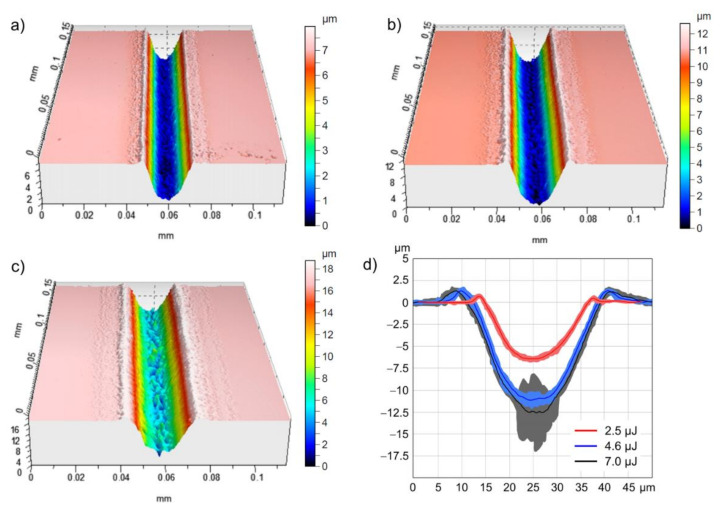
WLIM micrographs of the stainless-steel surface illustrating the 3D geometry of channel-like structures obtained from 10 over-scans with v = 0.1 m/s using the squared top-hat profile at z = 0 mm and different single-pulse energies: (**a**) E_imp_ = 2.5 µJ, (**b**) E_imp_ = 4.6 µJ and (**c**) E_imp_ = 7 µJ. A comparison between the cross-sections of the channels is illustrated in (**d**). Here, the solid line corresponds to the mean profile and the shadow illustrates the variation of the profile series measured along the channel.

These findings can also be observed in Figure 8, which shows 3D-WLIM micrographs of channels produced with the top-hat profile using different pulse energies, E_imp_. It becomes evident for moderate pulse energies that the homogeneous fluence distribution across the beam profile leads to a very high quality of both the walls and the bottom of the channel (Figure 8a,b). The corresponding arithmetical roughness values of the channel surface were measured via WLIM to be S_a_ = 0.1072 µm for E_imp_ = 2.5 µJ and S_a_ = 0.1844 µm for E_imp_ = 4.6 µJ. As indicated by the cross-sections in Figure 8d, the corresponding depth of the channels increases with increasing pulse energy, from about 6.2 µm at E_imp_ = 2.5 µJ (Figure 8a) to about 12.5 µm at E_imp_ = 7 µJ (Figure 8c). In addition, a slight increase in the channel width can be observed. However, the WLIM micrographs indicate that at very high pulse energies and thus large laser peak fluences, an inhomogeneous and rough channel bottom results from extensive ablation and the melt formation already detected in the single-spot experiments. The irregularity is expressed in particular in hole-like depressions that are not adequately displayed by the perspective of the 3D micrograph in Figure 8c. However, a qualitative measure is given by the grey-shaded area in the cross-section of Figure 8d that illustrates the remarkably larger variation, especially at the channel bottom, when compared to smaller E_imp_. This is also confirmed by the larger surface roughness that was measured to be S_a_ = 0.5174 µm for E_imp_ = 7 µJ.

**Figure 9:**
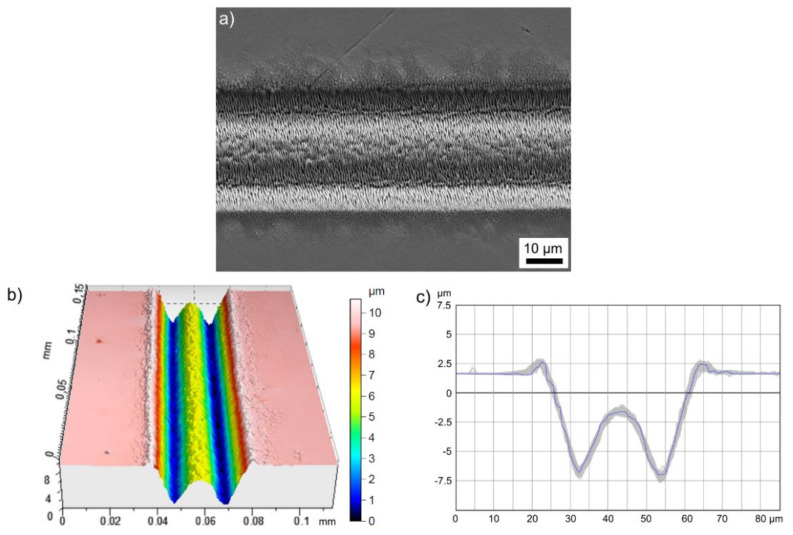
Micro-channel fabricated on stainless-steel using the squared donut-like intensity distribution at z = −0.9 mm, 10 over-scans with v = 0.1 m/s and a single-pulse energy of 4.6 µJ: (**a**) SEM micrograph, (**b**) WLIM micrograph and (**c**) cross-sectional height profile. The blue line in (**c**) corresponds to the mean profile and the grey shadow illustrates the profile series measured along the channel in (**b**).

Structuring with the donut-shaped intensity profile at z = −0.9 mm allows to create channels with steep walls and a w-shaped cross-sectional geometry that is determined by the specific intensity distribution (see Figure 5). According to the cross-sectional height profile of the channel (Figure 9c), 10 over-scans with v = 0.1 m/s and E_imp_ = 4.6 µJ result in a width of about 40 µm and a maximum ablation depth of about 10 µm. The channel walls are characterized by their very homogeneous and regular surfaces in the form of superimposed nanoscale LIPSS (Figure 9a). The high quality is confirmed by the fact that the modulation depth of LIPSS is typically in the order of 200 nm [30]. Contrary to a rotationally symmetrical donut, the used squared intensity profile provides the zero-intensity center and two axes in x- and y-directions with almost zero intensity. Consequently, the areas with lower (zero) intensity are not passed over by highly intensive beam parts during scanning in these directions, resulting in a channel with a less ablated central area (Figure 9b). This could be used, for example, to periodically propagate these w-shaped structures to large surface areas based on a suitable scan line distance. In dependence on the focal length of the focusing optics, this would result in a microscale periodic grating with periods in the range of 10–20 µm. Similar surface structures with comparable structural sizes can be obtained, e.g., from Direct Interference Laser Patterning, which is used among others to generate surfaces with functional properties (optics, tribology, biology, wetting) [32].

Generally, the results show that the donut and top-hat profiles provided by the beam-shaping element are advantageous for realizing micro-channels with specific wall inclination angles as well as tailor-made cross-sectional geometry. This option is also available (in situ) during the structuring process, since the individual intensity profiles can be addressed in a relatively simple way by changing the z-position of the sample surface.

## 4. Conclusions

The present study deals with a versatile refractive beam shaper that allows to convert the initial Gaussian intensity distribution of the fs-laser beam into different squared beam profiles at different z-positions. Numerical simulations for the beam-shaping unit were verified experimentally by spatial beam profile measurements and by surface structuring on the micro- and nano-scale. It was demonstrated that the squared top-hat profiles available in the focal region are advantageous to significantly reduce the processing time during large-area LIPSS structuring, while preserving their high regularity. Using the beam shaper for the generation of channel-like structures via multiple line scans, it was shown that the different focal intensity distributions resulted in precise channels with a controllable cross-sectional geometry.

## Data Availability

The data underlying this article will be shared on reasonable request from the corresponding author.
